# Supplementary material for: Drug repurposing for aging research using model organisms
Source: Aging Cell. 2017 Jun 16;16(5):1006–15. doi: 10.1111/acel.12626 (PMC5595691; doi:10.1111/acel.12626)
Supplement: Supplementary file 7 — Data S1 Zip‐Archive of all report cards. [file ACEL-16-1006-s007.zip › RC_NOH.pdf]

NOH

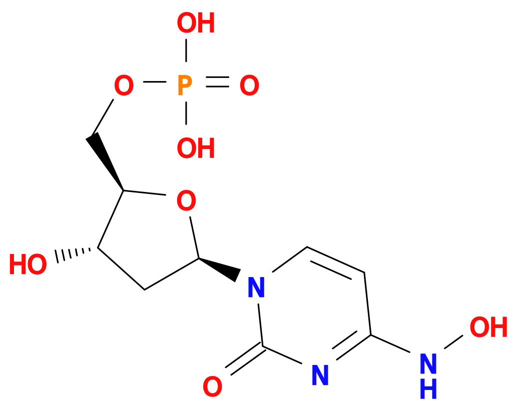

Database identifiers
ChEMBLCompound CHEMBL1161227

Ranking

	Rank	Score
Drosophila	481/697	0.293
C. elegans	543/591	0.034

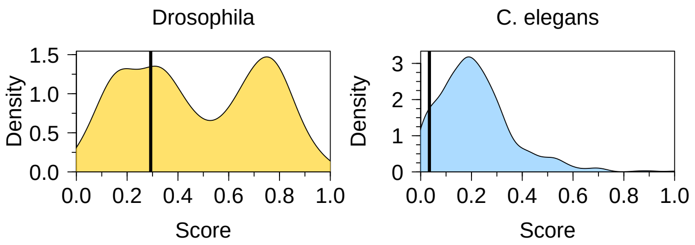

	Ageing implication	Domain conservation	Binding site conservation	Binding affinity	Bioavailability	Lipinski	Promiscuity	Purchasability	Drug approval	**Total**
Drosophila	0.792	0.932	0.96	0.538	(0.9)	-0.05	-0.0	0.0	0.0	0.293
C. elegans	0.792	0.922	1.0	0.538	0.216	-0.05	-0.0	0.0	0.0	0.034

Names

No synonyms found

Roles

ChEBI entry None has no roles

Status

Approved drug (according to ChEMBL)	No
Number of Rule of 5 violations	1
Binding affinity to original target in log units (RF-Score prediction)	5.15
Burns *C. elegans* bioavailability prediction	-4.8

Compound Target Characteristics

Thymidylate synthase

Best gene implication in ageing for this target family came from gene P45352 annotated in UniProt release 2014_02. Annotation GO 7568 (aging) was Inferred from Expression Pattern

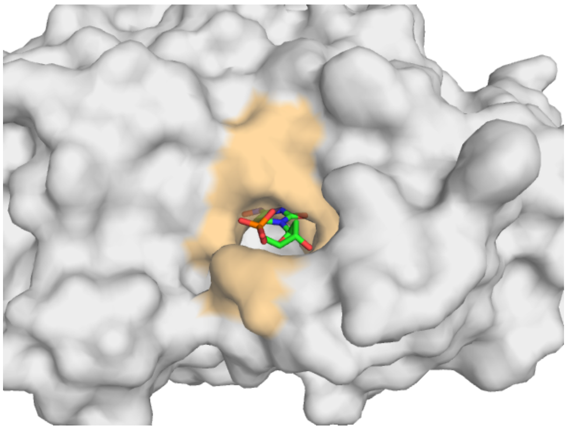

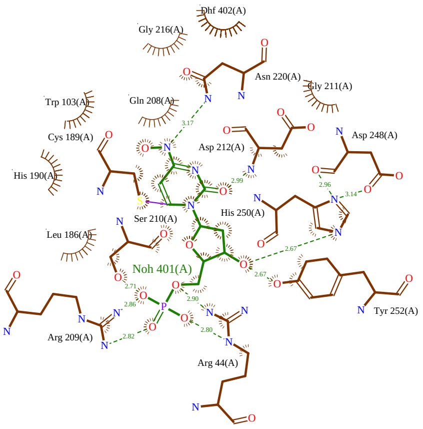

protein	amino acids contacts (binding site)													
PDB:4ez8:chainA:P07607	R	W	L	C	H	Q	R	S	G	D	G	N	H	Y
tr:Q53Y97:Q53Y97_HUMAN	R	W	L	C	H	Q	R	S	G	D	G	N	H	Y
sp:P04818:TYSY_HUMAN	R	W	L	C	H	Q	R	S	G	D	G	N	H	Y
sp:P45352:TYSY_RAT	R	W	L	C	H	Q	R	S	G	D	G	N	H	Y
sp:P07607:TYSY_MOUSE	R	W	L	C	H	Q	R	S	G	D	G	N	H	Y
tr:Q544L2:Q544L2_MOUSE	R	W	L	C	H	Q	R	S	G	D	G	N	H	Y
tr:Q541C9:Q541C9_DROME	R	W	L	C	H	Q	R	S	A	D	G	N	H	Y
sp:O76511:TYSY_DROME	R	W	L	C	H	Q	R	S	A	D	G	N	H	Y
tr:Q9Y052:Q9Y052_CAEEL	R	W	L	C	H	Q	R	S	G	D	G	N	H	Y
tr:Q9N588:Q9N588_CAEEL	R	W	L	C	H	Q	R	S	G	D	G	N	H	Y
sp:P06785:TYSY_YEAST	R	W	L	C	H	Q	R	S	C	D	G	N	H	Y

	whole protein		domain-based		contact-based	
protein	ident	simil	ident	simil	ident	simil
PDB:4ez8:chainA:P07607	1.0	1.0	1.0	1.0	1.0	1.0
tr:Q53Y97:Q53Y97_HUMAN	0.88	0.95	0.91	0.96	1.0	1.0
sp:P04818:TYSY_HUMAN	0.88	0.95	0.91	0.96	1.0	1.0
sp:P45352:TYSY_RAT	0.97	0.99	0.98	1.0	1.0	1.0
sp:P07607:TYSY_MOUSE	1.0	1.0	1.0	1.0	1.0	1.0
tr:Q544L2:Q544L2_MOUSE	1.0	1.0	1.0	1.0	1.0	1.0
tr:Q541C9:Q541C9_DROME	0.62	0.84	0.65	0.86	0.93	0.96
sp:O76511:TYSY_DROME	0.62	0.84	0.65	0.86	0.93	0.96
tr:Q9Y052:Q9Y052_CAEEL	0.6	0.81	0.63	0.85	1.0	1.0
tr:Q9N588:Q9N588_CAEEL	0.59	0.8	0.63	0.85	1.0	1.0
sp:P06785:TYSY_YEAST	0.58	0.79	0.61	0.83	0.93	0.89

Ts (FBgn0024920) associated phenotypes

neuroanatomy defective

(Information from FlyBase)

Ts (UniProt:O76511) annotation

Pathway: Pyrimidine metabolism; dTTP biosynthesis.
Subunit: Homodimer.

(Information from UniProt)

WBGene00022455 associated phenotypes

lethal, sterile

(Information from WormBase)
